# Elucidating the role of DEPTOR in Alzheimer’s disease

**DOI:** 10.3892/ijmm.2014.1895

**Published:** 2014-08-12

**Authors:** JULIE DAVIES, ELENA ZACHARIADES, KARLY-RAI ROGERS-BROADWAY, EMMANOUIL KARTERIS

**Affiliations:** Department of Biosciences, School of Health Sciences and Social Care, Brunel University, Uxbridge, UB8 3PH, UK

**Keywords:** mechanistic target of rapamycin, DEP domain-containing mTOR-interacting protein, Aβ peptide, Alzheimer’s disease

## Abstract

The mammalian or mechanistic target of rapamycin (mTOR) is a Ser/Thr protein kinase that, in response to nutrient stimulation, regulates cellular growth, proliferation, survival, protein synthesis and gene transcription. It has also been implicated in Alzheimer’s disease (AD) with neuronal cells and hippocampal slices of AD transgenic mice experiencing dysregulated mTOR and synaptic plasticity in response to treatment with the toxic amyloid β (Aβ^1–42^) peptide, which has been implicated in AD. DEP domain-containing mTOR-interacting protein (DEPTOR) is a protein which can bind to mTOR and cause its inhibition, and functions as a regulatory protein of mTOR to control its activity. The inhibition of mTOR has been shown to have a neuroprotective effect; in an animal model, it was shown to protect against Aβ-induced neurotoxicity. In the present study, to investigate to role of DEPTOR in a model of AD, we neuronally differentiated the SH-SY5Y cell line and examined the effects of treatment with an Aβ^42^ peptide, thus mimicking plaque formation. This resulted in a significant increase in mTOR and a significant decrease in DEPTOR expression compared to the unstimulated controls. Moreover, to the best of our knowledge, we demonstrate for the first time a reduction in the protein level of DEPTOR in the precentral gyrus, postcentral gyrus and occipital lobe of a brain with AD compared to a normal control, as well as a significant reduction in DEPTOR expression in samples from late-onset AD (LOAD) compared to early-onset familial AD (EOFAD). The reduction in DEPTOR expression in cases of AD compared to healthy controls can lead to an augmentation of mTOR signalling, leading to Aβ accumulation, which in turn leads to a further reduction in DEPTOR expression. This results in the accumulation of amyloid plaque, shifting the balance from neuroprotection to neurodegeneration.

## Introduction

The pathogenesis of Alzheimer’s disease (AD) is multi-aetiological, and it is likely due to these various origins that AD induces diverse neuropathological changes. The most prominent lesions in brains with AD are atrophy, a large number of senile plaques (SPs) formed by amyloid β (Aβ) between neurons and neurofibrillary tangles (NFTs) made of abnormally hyperphosphorylated tau protein in neurons (1). Several other mechanisms have also been proposed to explain the pathogenesis of AD. Although each of these mechanisms may contribute to the pathogenesis of the disease, the extent to which they drive the neurodegenerative process is uncertain (2). This is further complicated by the fact that two different pathways are detected in neurons, i.e., neurodegeneration and neurofibrillary degeneration. Neurodegeneration incorporates different abnormal signalling pathways that lead to neuronal loss, including apoptosis and other modes of cell death. Neurofibrillary degeneration is a specific type of neuronal reaction marked by the accumulation of hyperphosphorylated tau protein as paired helical filaments in NFTs, which can also induce abnormal neuronal metabolism and death. Of note, these two pathways share mTOR-dependent signalling as a common key regulator (1).

The mammalian or mechanistic target of rapamycin (mTOR) is a Ser/Thr protein kinase that functions as an adenosine triphosphate (ATP) and amino acid sensor to balance nutrient availability and cell growth (3,4). mTOR is capable of forming two complexes named mTORC1 and mTORC2 (5). The rapamycin-sensitive mTORC1 contains the following proteins: raptor, GβL (also known as mLST8) and the proline-rich Akt substrate of 40 kDa (PRAS40). The mTORC2 complex contains rictor, mamalian stress-activated protein kinase (SAPK)-interacting protein 1 (mSIN1), Protor-1 and GβL5 (5). A number of recent studies have found a strong link between mTOR and AD. For example, mTOR is critical for long-lasting forms of synaptic plasticity and long-term memory (LTM) formation, which is impaired in mouse models of AD (6). The importance of mTOR in synaptic plasticity is in agreement with the central role of mTOR in controlling transcriptional events, since *de novo* protein synthesis is involved in these long-lasting forms of synaptic plasticity and LTM (7). The inhibition of the mTOR pathway appears to modulate the process of aging, a well-established risk factor for AD (8,9). Moreover, autophagy, a pathway for organelle and protein turnover, has been implicated in neurodegeneration. Autophagy is constitutively active and highly efficient in healthy neurons and rapamycin (an mTOR inhibitor) is able to induce autophagy (10). Finally, mTOR signalling has been shown to be altered in models of AD (11,12). mTOR signalling has been shown to be inhibited both in cultured neurons and hippocampal slices from AD transgenic mice and in wild-type (WT) hippocampal slices exposed to exogenous Aβ^1–42^, and this mTOR dysregulation correlates with impairment in synaptic plasticity (13).

Recently, a novel endogenous inhibitor of the mTOR pathway, termed DEP domain-containing mTOR-interacting protein (DEPTOR), has been shown to bind to both the mTORC1 and mTORC2 complexes (14). Its precise function has not yet been fully elucidated; however, knocking down DEPTOR leads to the activation of signalling through mTORC1 and mTORC2. This is demonstrated both by the observation that there is a change in the phosphorylation status of S6 kinase 1 (S6K1) and protein kinase B (PKB) when DEPTOR levels are decreased (by RNA-based interference) and by the increased *in vitro* activity against these substrates of mTOR complexes from cells with decreased levels of DEPTOR (14,15). Moreover, DEPTOR has been shown to be downregulated in malignancies of the prostate, bladder, head and neck, cervix and thyroid (14), whereas we have previously demonstrated the significant upregulation of DEPTOR in two paclitaxel-resistant ovarian cancer cell lines when compared to the parental ones (16). Evidence of the potential involvement of DEPTOR in AD arises from studies in which resveratrol (RSV), a naturally occurring polyphenol, has been used. RSV inhibits mTOR signalling by promoting the interaction between mTOR and DEPTOR *in vitro* (17). Recent studies have indicated that RSV has neuroprotective properties (18,19). In an animal model, RSV has been shown to protect rats from Aβ-induced neurotoxicity (20).

Emerging data suggest that the augmentation of mTOR signalling is involved in the aetiopathogenesis of AD. We hypothesised that DEPTOR is an integral modulator of both the mTORC1 and mTORC2 complexes, and the presence or degree of binding to the complexes may determine at which point mTOR will be inhibited/activated, thus leading to a neuroprotective or neurotoxic effect.

## Materials and methods

### Cell culture

SH-SY5Y cells (ATCC; Manassas, VA, USA) were cultured in 1:1 EMEM and Hams F12 (Sigma-Aldrich, Gillingham, UK) supplemeted with 10% FBS (50 ml; Gibco, Paisley, UK), 1% non-essential amino acids, 1% 200 mM L-glutamine, 1% penicillin/streptomycin (Gibco) at 37°C with 5% CO_2_. The SH-SY5Y cells were neuronally differentiated for 6 days by treatment with 10 μM retinoic acid (RA) (Sigma-Aldrich).

### Quantitative RT-PCR

Anterior hippocampus with entorhinal cortex samples from adults with AD [n=10; 5 with early-onset familial AD (EOFAD); median age, 61 years; and 5 with late-onset AD (LOAD); median age, 84 years] were provided in collaboration with Brains for Dementia Research (BDR), University of Newcastle, Newcastle upon Tyne, UK. The relative expression of the genes of interest was assessed by quantitative PCR (qPCR) on an ABI PRISM^®^ 7900HT Sequence detection system (Applied Biosystems, Foster City, CA, USA) using SYBR^®^-Green PCR reaction mixture (Sigma-Aldrich) and the primers for mTOR and DEPTOR as previously described (21). As a negative control for all the reactions, distilled water was used in place of the cDNA. RNAs were assayed from 3 independent biological replicates. The RNA levels were expressed as a relative quantification using the housekeeping gene, 18S rRNA (RQ) value. The ΔCt method was employed for comparing relative expression results between treatments in qPCR, as previously described (22).

### Western blot analysis

Protein lysates in 1X Laemmli buffer (Sigma-Aldrich) were separated on an SDS-10% polyacrylamide gel (Sigma-Aldrich) and the proteins were transferred onto nitrocellulose membranes (GE Healthcare, Buckinghamshire, UK). The membranes were blocked in TBS (Fisher Scientific, Loughborough, UK) containing 5% dried milk powder (w/v) and 0.1% Tween-20 (Fisher Scientific), for 1 h at room temperature. Following 3 washes with TBS-0.1% Tween-20, the nitrocellulose membranes were incubated with primary antibodies against Aβ^42^ and GAPDH (both from Cell Signalling Technology, Danvers, MA, USA). The primary antisera were used at a 1:1,000 dilution overnight at 4°C. The membranes were washed thoroughly for 30 min with TBS-0.1% Tween-20 prior to incubation with the secondary antibody, HRP-conjugated immunoglobulin (1:2,000; Sigma-Aldrich), for 1 h at room temperature and further washing for 30 min with TBS-0.1% Tween-20. Antibody complexes were visualised as previously described (21).

### Immunofluorescence staining of AD sections

Three brain regions (precentral gyrus, postcentral gyrus and occipital lobe) from a single brain with AD and a normal brain were obtained as a tissue microarray from LifeSpan Biosciences Inc. (LSBio; Seattle, WA, USA). The ages of the patients were 75 and 54 years, respectively, and they were both male. Following a series of deparaffinisations and dehydrations, the slides were incubated with 10% bovine serum albumin (BSA; Sigma-Aldrich) for 1 h. This was followed by incubation for 1 h with an antibody against DEPTOR (Millipore, Abingdon, UK), at a 1,200 dilution in 1% BSA/PBS. The cells were then washed with PBS prior to an incubation with a fluorescein isothiocyanate (FITC)-conjugated secondary antibody (Santa Cruz Biotechnology, Inc., Santa Cruz, CA, USA) for 1 h. The slides were washed with PBS and mounted in VECTASHIELD^®^ Mounting Medium (Vector Laboratories, Inc., Burlingame, CA, USA) containing the dye, 4,6-diamido-2-phenylindole (DAPI) to counterstain the nuclei. Images were captured using a Plan Apo Neofluar 63X NA 1.25 oil objective (Zeiss, Thornwood, NY, USA) on a Zeiss Axiovert 200 M microscope and viewed using AxioVision software. The images were then analyzed using ImageJ 1.34 image analysis software (National Institutes of Health, Bethesda, MD, USA).

### Immunofluorescence staining of SH-SY5Y cells

The neuronally differentiated SH-SY5Y cells were fixed in 4% paraformaldehyde (Sigma-Aldrich) for 10 min prior to washes in PBS and incubation with 10% BSA for 1 h. The cells were incubated for 1 h with DEPTOR (Millipore), mTOR (Cell Signalling Technology) and pan-neuronal marker (Millipore) antibodies at a 1:100 dilution in 1% BSA/PBS. The cells were then washed with PBS prior to a further incubation with secondary antibodies as previously described (21). Images were captured using a Plan Apo Neofluar 63X NA 1.25 oil objective (Zeiss) on a Zeiss Axiovert 200 M microscope and viewed using AxioVision software.

### Statistical analysis

qPCR data are reported as the means ± standard error of the mean (SEM). Statistical analysis was performed using the Student’s t-test. A value of p<0.05 was considered to indicate a statistically significant difference.

## Results

### Development of an in vitro neuronal model

**Figure 1:**
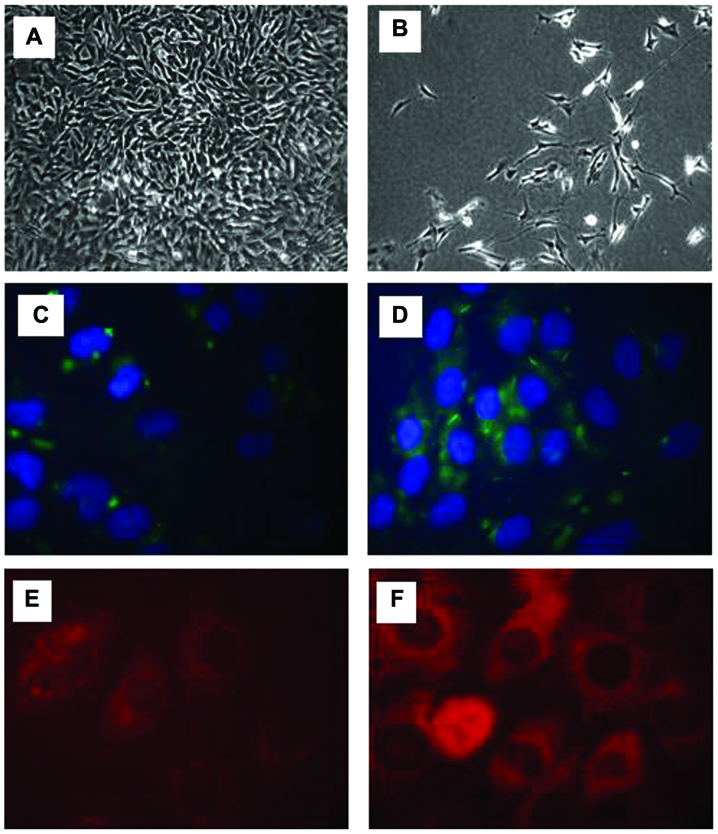
Retinoic acid (RA)-induced differentiation of SH-SY5Y cells for 6 days with 10 μM RA. (A) Cell microscopy of undifferentiated cells and (B) differentiated cells. Immunofluorence staining of cells using a PanNeuronal Marker (C) before and (D) after neuronal differentiation. Mechanistic target of rapamycin (mTOR) expression (E) shown by immunofluorence staining and (F) with a DEPTOR antibody in differentiated SH-SY5Y cells.

The morphological changes of the SH-SY5Y were monitored during the differentiation process using a microscope. The SH-SY5Y cells were seeded at 1×10^6^ and the undifferentiated SH-SY5Y cells were fast-growing and rounded in shape (Fig. 1A). Following differentiation, the cells did not reach confluence and by day 6, neurite extensions were prominent (Fig. 1B). A pan-neuronal marker was then used in the differentiated and undifferentiated cells in order to observe the changes occurring in fundamental somatic, nuclear, dendritic and axonal proteins. In the undifferentiated cells, there was a low expression of neuronal proteins, as SH-SY5Y is a neuroblastoma cell line (Fig. 1C). Following differentiation using RA for 6 days, the SH-SY5Y cells demonstrated a notable increase in the pan-neuronal marker signal, further confirming the acquisition of a neuronal phenotype (Fig. 1D). We then assessed the protein expression of mTOR and DEPTOR in the neuronally differentiated cells. Using immunofluorescence, an intense cytoplasmic staining was observed for both mTOR (Fig. 1E) and DEPTOR (Fig. 1F).

### Treatment of differentiated SH-SY5Y cells with Aβ^42^ to mimic an AD milieu in vitro: effects on mTOR and DEPTOR

**Figure 2:**
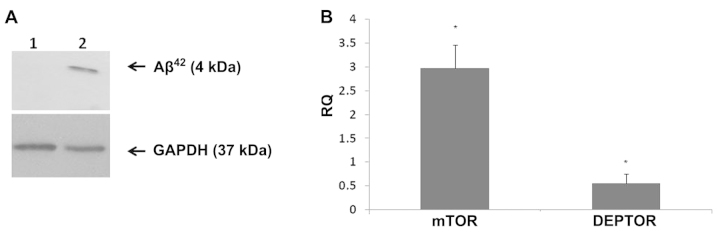
Differentiated SH-SY5Y cells were treated with an amyloid β *(*Aβ)^42^ peptide. (A) Western blot analysis of extracted protein revealed that, in the control (lane 1), there was no Aβ^42^ deposition, but with Aβ^42^ treatment there was an accumulation of the peptide detected at 4 kDa (lane 2). (B) Quantitative polymerase chain reaction (qPCR) of cells treated with Aβ^42^ revealed a significant increase in mechanistic target of rapamycin (mTOR) expression compared to the untreated cells and a significant reduction in DEPTOR expression over 24 h. Data are the means ± standard error of the mean (SEM). ^*^P<0.05, stastistically significant difference compared to the untreated control samples (n=3).

As already mentioned in the Introduction, one of the hallmarks of AD is the large number of SPs formed by an accumulation of toxic Aβ between neurons. Aβ^42^ in patients with AD has been shown to reach concentrations lower than 10 nM, but can reach μM ranges (23–25). Our aim was to treat fully differentiated SH-SY5Y cells with 1 μM Aβ^42^ in an attempt to mimic an AD milieu and assess its effects on mTOR and DEPTOR expression *in vitro*. Western blot analysis was performed to ensure that Aβ^42^ was being deposited. In the cells treated with Aβ^42^, there was protein deposition which was detected at 4 kDa (Fig. 2A). Fully differentiated SH-SY5Y cells were seeded at a density of 1×10^5^/well, serum-starved for 4 h and treated with Aβ^42^ (1 μM) for 24 h. mTOR epxression was markedly increased (2-fold) upon 24 h of Aβ^42^ treatment, whereas DEPTOR expression was markedly decreased compared to the control (Fig. 2B).

### Differential expression of DEPTOR in brains with AD

**Figure 3:**
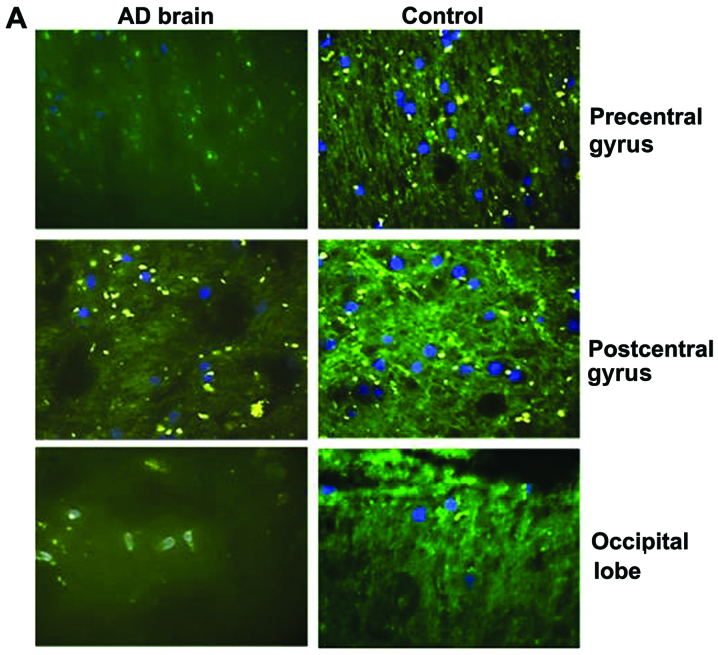

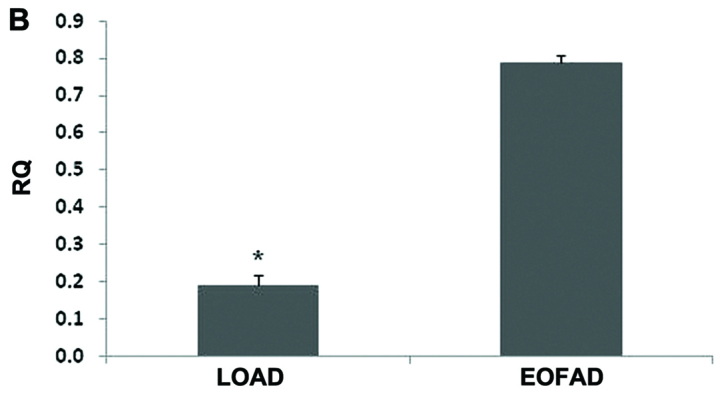
(A) Immunohistochemical staining with a DEPTOR antibody revealed a reduction in its expression in the precentral gyrus, postcentral gyrus and occipital lobe of a brain with Alzheimer’s disease (AD) compared to a healthy control brain. (B) DEPTOR expression levels in human late onset AD (LOAD) samples were significantly reduced compared to early onset familial AD (EOFAD) samples. Data are the means ± standard error of the mean (SEM). ^*^P<0.05, stastistically significant difference between LOAD and EOFAD (n=5).

We then assessed the protein expression of DEPTOR from 3 brain regions from a single brain with AD and a normal (healthy) brain. The ages of the patients were of 75 and 54 years, respectively, and they were both male. In all 3 different regions, i.e., precentral gyrus, postcentral gyrus and occipital lobe, the expression of DEPTOR was markedly decreased in the patient with AD compared to the same region of the healthy control (Fig. 3A).

Using clinical samples from patients with EOFAD (median age, 61 years) and LOAD (median age, 84 years) we examined the expression of DEPTOR at the mRNA level using qPCR. There was a significant (~4-fold) decrease in the expression of DEPTOR in the patients with LOAD when compared with the patients with EOFAD (Fig. 3B).

## Discussion

To the best of our knowledge, in this study, we demonstrate for first time the expression of DEPTOR in human brains, as well as the mechanisms through which its expression is affected by Aβ^42^ accumulation. Several disease mechanisms involved in AD affect the structure, as well as the signalling experienced by neurons; thus, it is essential to obtain a model that is more similar to an *in vivo* situation so as to study more comparable results. Immortalised cell lines are an appropriate solution to study this; however, they lack some of the key characteristics of human neurons, such as morphology, cessation of mitosis and the expression of neuronal markers. The transition to a more neuronal phenotype is vital for mimicking a disease or a signalling milieu *in vitro*. Due to extensive study previously performed on SH-SY5Y cells, combined with the comprehensive use of this cell line in previous research on AD, we decided that this would be the most appropriate cell line to use (26,27). Neuronal differentiation was accomplished by the addition of RA and validated by observing cell morphology, neurite extension and the expression of neuronal proteins. In this model, both mTOR and DEPTOR appeared to be expressed at the protein level, mainly localised in the cytoplasm. This finding corroborates findings from previous studies performed in our laboratory, demonstrating that the cellular distribution of mTOR is primarily cytoplasmic (21). mTOR can also shuttle between the nucleus and cytoplasm (28), and in the SH-SY5Y cells, DEPTOR was also primarily localised in the cytoplasm; however, there was some immunofluorescence staining in the nucleus. This could be suggestive of potential trafficking to the nucleus alone or as part of the mTORC1 complex.

As already mentioned, one of most important hallmarks of AD is the accumulation of Aβ^42^, causing the formation of Aβ plaques. When the SH-SY5Y cells were treated with the ‘toxic’ Aβ^42^ peptide, this led to a decrease in the expression of DEPTOR and a marked upregulation in mTOR gene expression. This is the first time that Aβ^42^ appears to differentially affect key mTOR signalling components *in vitro*. We then expanded our observations using clinical samples, where there was a notable decrease in DEPTOR immunostaining in certain regions of the brain with AD when compared to a healthy control. This is of increasing importance, as microstructural damage to the precentral gyrus of patients with AD, as well as a thinning of the postcentral gyrus, have been recently demonstrated (29,30). It is also thought that damage incurred by the occipital lobe in AD can greatly contribute to psychotic symptoms, as a result of deficits in attention and visual systems (31,32).

We also provide novel data regarding the differential expression of DEPTOR in early- and late-onset AD. It is evident that, in LOAD, there is a marked downregulation in DEPTOR expression, in agreement with the diminished expression of this protein in brain regions with AD. EOFAD presents before 65 years of age and there are multiple cases within a family usually attributable to mutations in the amyloid precursor protein (*APP*) gene, the presenilin (*PSEN)1* gene or the *PSEN2* gene (33). By contrast, LOAD occurs after 65 years of age and is sporadic with no known single targetable cause (34). We would like to acknowledge that, due to ethical restrictions, we were unable to obtain more brain samples in order to investigate the expression and cellular distribution of other components of the mTOR signalling pathway.

Our study provides new insight into the higher order of complexity regarding the involvement of DEPTOR and mTOR in the aetiopathogenesis of AD. Caccamo *et al* (35) demonstrated that Aβ accumulation causes mTOR hyperactivity by regulating PRAS40 phosphorylation, whereas RSV promotes the non-amyloidogenic cleavage of the amyloid precursor protein, the enhanced clearance of Aβ-peptides and reduced neuronal damage (19). Of note, RSV exerts its effects by tightly complexing DEPTOR with mTOR. DEPTOR depletion enhances the *in vitro* kinase activity of the mTORC1 and mTORC2 complexes. More specifically, mTORC1 immunopurified from cells depleted of DEPTOR has increased *in vitro* kinase activity towards two known substrates, S6K1 and 4EBP1. Of note, these molecules control the synthesis of β- or α-secretase and, therefore, Aβ generation (36).

Treatment of differentiated mouse N2a neuroblastoma cells with Aβ^42^ has been shown to induce the phosphorylation of mTOR at S2448 and of p70S6K at T38927. Similarly, in differentiated human SH-SY5Y neuroblastoma cells, a significant upregulation of p-p70S6K at T389 and T421/S424 sites was induced following treatment with Aβ^42^ (11).

**Figure 4:**
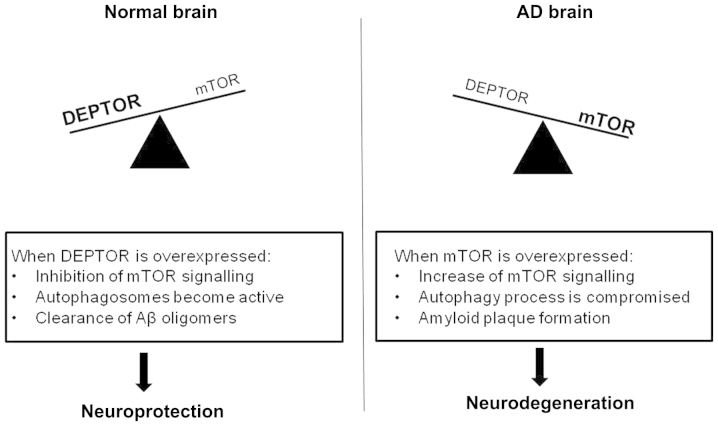
Proposed model for the interaction of the mechanistic target of rapamycin (mTOR)/DEPTOR in a normal brain and a brain with Alzheimer’s disease (AD).

Taken together, we would like to propose the following (Fig. 4) that builds upon the model proposal shown in the study by Cai *et al* (36): under normal conditions, mTOR activity is modulated by DEPTOR. This will allow autophagosomes to clear any toxic Aβ oligomers, thus favouring a neuroprotective environment. In patients with AD, the mTOR pathway can be overactive, leading to a dysfunction of autophagy and, consequently, a lack of Aβ clearance. In turn, the excess of Aβ will cause a further decrease in DEPTOR and an increase in mTOR expression, augmenting the neurodegenerative process.
